# The biological function of the long non-coding RNA endogenous born avirus-like nucleoprotein in lung adenocarcinoma is mediated through the microRNA-655-3p/B-cell lymphoma-2 axis

**DOI:** 10.1080/21655979.2022.2065946

**Published:** 2022-04-27

**Authors:** Xiaopeng Wang, Jing Yin

**Affiliations:** a Department of Respiratory and Critical Care Medicine, Puren Hospital Affiliated to Wuhan University of Science and Technology, Wuhan, China; b Department of Respiratory and Critical Care Medicine, Renmin Hospital of Wuhan University, Wuhan, China

**Keywords:** LncRNA EBLN3P, miR-655-3p, Bcl-2, lung adenocarcinoma, apoptosis

## Abstract

Lung adenocarcinoma (LUAD) is a subtype of lung cancer, and therapy remains a great challenge. A growing body of evidence shows that long-chain non-coding RNAs (lncRNAs) play an important role in the occurrence and development of LUAD. This study investigated the roles and mechanisms of action of EBLN3P in LUAD. The bioinformatics software starBase and TargetScan were used to predict the binding sites of the lncRNA endogenous born avirus-like nucleoprotein (EBLN3P) and microRNA (miR)-655-3p in LUAD. The regulatory role of EBLN3P and miR-655-3p in cell proliferation was verified through the 3-(4,5-dimethylthiazol-2-yl)-2,5-diphenyl-2 H-tetrazolium bromide (MTT) assay. The binding sites between EBLN3P, miR-655-3p, and B-cell lymphoma-2 (Bcl-2) were assessed using dual-luciferase reporter assay, western blotting, and quantitative reverse transcription polymerase chain reaction (qRT-PCR). Flow cytometry (FCM) was performed to analyze the apoptotic rates of A549 cells after transfection. The results revealed that EBLN3P was upregulated, whereas miR-655-3p was downregulated in LUAD cell lines (A549 and NCI-H23). Bioinformatics analysis and dual-luciferase reporter assays indicated that EBLN3P interacted with miR-655-3p. Knockdown of EBLN3P notably inhibited the bioactivity and induced apoptosis in A549 cells by upregulating miR-655-3p. Mechanistically, miR-655-3p inhibits cell viability and induces apoptosis by inhibiting Bcl-2 expression. The high expression of Bcl-2 reversed the impact of miR-655-3p on the inhibition of cell bioactivity and induction of apoptosis in A549 cells. In conclusion, this study demonstrated that EBLN3P silencing inhibits bioactivity and induces apoptosis via the miR-655-3p/Bcl-2 axis, providing a potential therapeutic target for lung adenocarcinoma.

## Graphical abstract

**Figure:**
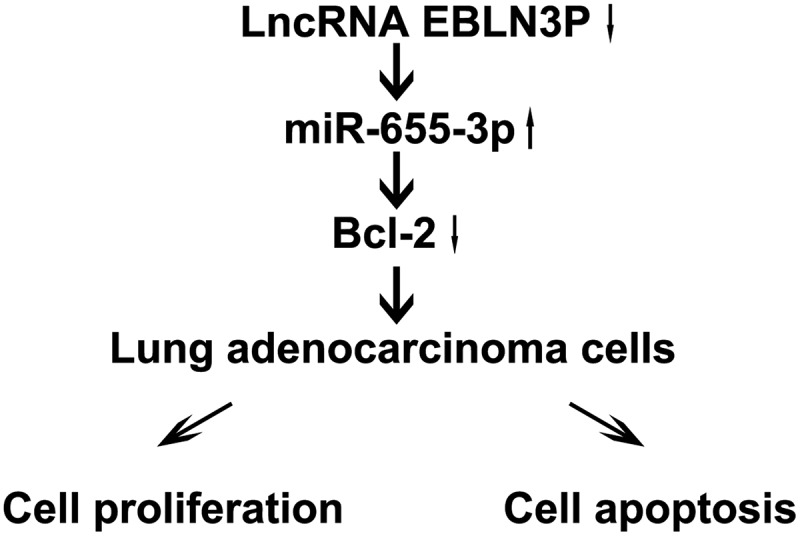

## Introduction

Lung adenocarcinoma (LUAD) is the most common cause of cancer-related deaths worldwide [1]. Although there have already been improvements in early diagnosis through newly developed therapies and technologies, many patients with LUAD are usually informed when they already have advanced disease, ultimately displaying a low survival rate [2,3]. Cancer biomarkers are critical tools for preventing malignancies, but there are currently few established biomarkers for clinical treatment [4]. Therefore, elucidating the pathogenesis of LUAD and identifying new biomarkers are essential for the treatment of LUAD.

Long non-coding RNAs (lncRNAs) are RNA molecules with a transcription length of over 200 nt [5]. Studies have found that lncRNAs regulate gene expression via epigenetic, transcriptional, and post-transcriptional regulation but do not encode proteins [6]. In addition, several studies have shown that lncRNAs are specifically expressed in a majority of tumors, exhibiting anticancer effects [7]. Moreover, lncRNAs act as precursors of competitive endogenous RNAs (ceRNAs) to regulate cell fate and affect gene silencing by binding to microRNAs (miRNAs) [8]. It has been confirmed that the relationship between HER2 and miR-331-3p may regulate the roles of HOTAIR in gastric cancer [9]. Study has found that lncRNA PCAT1 binds to miRNAs to regulate cell proliferation in esophageal squamous cell carcinoma [10]. LncRNAs have also been extensively studied in LUAD [11,12]. LncRNA MANCR (mitosis-related lncRNA, LINC00704) can lead to enhanced proliferative, invasive and migratory abilities of LUAD cells while reducing cell apoptosis [13]. LncRNA MSC-AS1 (musculin antisense RNA 1) has been reported to facilitate LUAD through sponging miR-33b-5p [14]. LncRNA-LINC01089 has been found to repress LUAD cell proliferation and promote apoptosis via sponging miR-543 [15]. Endogenous bornavirus-like nucleoprotein (EBLN3P) is a recently discovered lncRNA [16]. Studies have found that EBLN3P promotes the occurrence and development of rectal cancer by targeting miR-323a-3p/UHMK1 [16]. In addition, EBLN3P could regulate the expression of Rab10 through competitive sponging action with miR-224-5p in osteosarcoma [17]. Similarly, EBLN3P influenced the progression of hepatocellular carcinoma (HCC) by regulating the expression of DOCK4 via miR-144-3p [18]. However, the regulatory mechanisms of EBLN3P in LUAD remain unclear.

MiRNAs are evolutionarily conserved non-coding small RNAs approximately 18–20 bp long [19]. The dysregulation of miRNAs can be used as tumor inhibitors, affecting cell viability, apoptosis, and the epithelial-mesenchymal transition (EMT) [20,21]. Therefore, lncRNAs and miRNAs can be effective biomarkers for tumor diagnosis. MiR-655-3p is an important component of the miRNA-regulated network. Recent studies have demonstrated that miR-655-3p plays a key regulatory role in several diseases by primarily inhibiting EMT through the ZEB1 and TGFBR2 axes [22]. Recent studies have found that miR-655-3p inhibits cell metastasis by targeting pituitary tumor transformation gene 1 (PTTG1) in the lung cancer cell line A549 [23]. Additionally, MALAT1 promotes ATAD2 expression through miR-655-3p to regulate the progression of retinoblastoma [24]. However, it is unclear whether miR-655-3p interacts with other lncRNAs such as EBLN3P to regulate the progression of LUAD.

Studies have found that miRNAs influence gene expression through mRNA-specific binding sites; Bcl-2 is a target gene of miRNA (such as miR-8, miR-153-3p) [25,26]. Research has shown that miR-15b targets Bcl-2 in LUAD to promote cell migration and EMT [27]. In addition, Bcl-2 is a downstream regulator of miR-153-3p and miR-136, regulating apoptosis in various cells [26,28]. However, the role of Bcl-2 and miR-655-3p in LUAD remains unclear.

Through bioinformatics software analysis, we found that there is a binding site between lncRNA-EBLN3P and miR-655-3p, and Bcl-2 is a potential target gene of miR-655-3p. Thus, in this study, we speculated that lncRNA-EBLN3P is involved in the progression of LUAD through the miR-655-3p/Bcl-2 axis. And this study was performed to explore the roles and potential mechanisms of EBLN3P in LUAD. Our results showed that EBLN3P could be used as a biomarker in patients with LUAD.

## Materials and Methods

### Cell Culture

LUAD cell lines (NCI-H23 and A549), a normal lung epithelial cell line (BEAS2B), and HEK-293 T cells were obtained from the American Type Culture Collection (ATCC, USA). All cells were cultured in Dulbecco’s modified Eagle’s medium (DMEM) containing 1% penicillin, streptomycin, and 10% fetal bovine serum (FBS) at 37°C in the presence of 5% CO_2_.

### qRT-PCR assay

Total RNA was extracted from the cells using TRIzol® reagent (Invitrogen, Carlsbad, CA, USA) according to the manufacturer’s instructions. cDNA was synthesized using a PrimeScript® RT Master Mix reagent kit (Takara). qRT-PCR was carried out using SYBR green reagents (Vazyme, Nanjing, China) to examine the relative mRNA levels of the indicated genes according to the following protocol: 95°C for 30s, followed by 45 cycles of 95°C for 5 s and 60°C for 1 min. U6 and GAPDH were used as internal references for miRNA and mRNA expressions, respectively. The expression of the target genes was verified using the 2^−ΔΔCt^ assay [29]. The primer sequences used are listed in Table 1.

**Table 1.** Primer Sequences for qRT-PCR

Gene	Forward sequence	Reverse sequence
EBLN3P	5’-CAGACTAAAGGATCAAGCGAGA-3’	5’-ATCAATTGCCACAGGTTGAAGA-3’
miR-655-3p	5’-CGCGCGATAATACATGGTTAAC-3’	5’-GTGTCTTAAGGCTAGGCCTA-3’
cyclinD1	5’-GGCGGAGGAGAACAAACAGA-3’	5’-ATGGAGGGCGGATTGGAAA-3’
Bax	5’-CCCGAGAGGTCTTTTTCCGAG-3’	5’-CCAGCCCATGATGGTTCTGAT-3’
Bcl-2	5’-GGTGGGGTCATGTGTGTGG-3’	5’-CGGTTCAGGTACTCAGTCATCC-3’
U6	5’-CTCGCTTCGGCAGCACA-3’	5’-AACGCTTCACGAATTTGCGT-3’
GAPDH	5’-CCAGGTGGTCTCCTCTGA-3’	5’-GCTGTAGCCAAATCGTTGT-3’

### Western blot analysis

Protein levels were determined using western blot assay [30]. Total protein was extracted from cells using a RIPA lysis buffer containing a protease inhibitor. Protein concentrations were then calculated using a BCA protein analysis kit (Invitrogen). Afterward, 25 μg of protein was isolated via SDS-PAGE and transferred onto nitrocellulose membranes (Millipore). After blocking with 5% nonfat milk for 2 h, the membranes were incubated with the following primary antibodies overnight at 4°C: anti-cyclin D1 (cat. no. 55,506; 1: 1000; Cell signaling Technology, Beverly, MA, USA), anti-Bax (cat. No. 5023; 1: 1000; Cell signaling Technology, Beverly, MA, USA), anti-Bcl-2 (cat. No. 4223; 1: 1000; Cell signaling Technology, Beverly, MA, USA), and anti-GAPDH (cat. No. 5174; 1: 1000; Cell signaling Technology, Beverly, MA, USA). The membranes were then washed with TBST and incubated with the appropriate secondary antibody (cat. No. 7074; 1: 2000; Cell signaling Technology, Beverly, MA, USA) for 4 h at 4°C. Finally, the blots were visualized using an enhanced chemiluminescence (ECL) reagent to detect protein expression.

### Plasmid construction and cell transfection

Small interfering RNA (siRNA) for EBLN3P (EBLN3P-siRNA) and its corresponding negative control (control-siRNA), miR-655-3p inhibitor, inhibitor control, miR-655-3p mimic, and mimic control were obtained from RiboBio (Guangzhou). The Bcl-2-plasmid and control-plasmid were obtained from Santa Cruz. Transfection was performed using Lipofectamine 6000 (Invitrogen) according to the manufacturer’s protocol [31]. The A549 cells were collected for further assays 48 h after transfection.

### Bioinformatic analysis

The complementary sequences between EBLN3P and miR-655-3p were predicted using Starbase (http://starbase.sysu.edu.cn/) [32]. Bcl-2 fragments containing miR-655-3p binding sites were predicted using TargetScan 7.0 (http://www.targetscan.org/vert_72/) [33].

### Dual-luciferase reporter assay

A dual-luciferase reporter assay was performed as reported previously [34]. Wild-type or mutant EBLN3P and Bcl-2 fragments containing miR-655-3p binding sites were synthesized and inserted into the pGL3-basic plasmid (Promega, Madison, WI, USA) to construct EBLN3P-WT or EBLN3P-Mut and Bcl-2-WT or Bcl-2-Mut. HEK 293 T cells were co-transfected with the corresponding reporter vectors and the miR-655-3p or mimic control. After 48 h of transfection, a dual-luciferase gene reporter system (Promega, USA) was used to detect luciferase activity.

### MTT assay

Cell viability was evaluated using the MTT assay, as described previously [35]. After transfection, 5 × 10^3^ cells were seeded into each well of a 96-well plate and cultured for 0, 24, 48, or 72 h before subjecting them to the MTT assay. At the indicated time points, the cells were incubated with MTT for 4 h at 37°C. The absorbance was measured at 570 nm using a microplate reader.

### Cell apoptosis analysis

The cell apoptosis rate was determined by staining the cells using an Annexin V-FITC/PI Apoptosis Kit (Beyotime) [36]. Briefly, the cells were harvested and incubated with 5 μL Annexin V-FITC reagent and 5 μL PI reagent at 37°C for 20 min in the dark. The cell apoptotic ratio was then analyzed using flow cytometry (Beckman, USA).

### Caspase-3 activity assay

Caspase-3 activity was measured using a Caspase-3 Activity Kit (Beyotime), and the values were measured using a microplate reader at 405 nm [37].

### Statistical analysis

All statistical analyses were performed using SPSS 20.0 software. All experiments were performed in triplicate, and the results are presented as the mean ± SD. Differences between two groups were compared using Student’s t-test and one-way ANOVA analysis followed by Tukey’s test were used for multiple groups. Statistical significance was set at *P* < 0.05.

## Results

### Expression level of EBLN3P and miR-655-3p in lung adenocarcinoma cells

**Figure 1.**
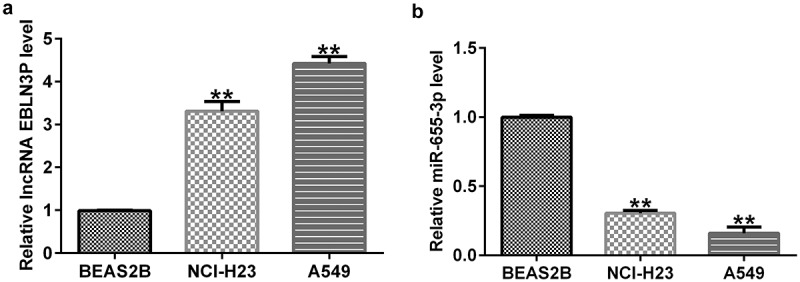
EBLN3P and miR-655-3p expression in lung adenocarcinoma cell lines.

To explore the effects of EBLN3P and miR-655-3p in LUAD, we first measured their expression levels in LUAD. First, we performed qRT-PCR to examine the levels of EBLN3P and miR-655-3p in the lung adenocarcinoma cell lines A549 and NCI-H23, as well as in BEAS2B cells. The RNA expression level of EBLN3P was increased in A549 and NCI-H23 versus BEAS2B cells (Figure 1a). In contrast, the data showed that the expression level of miR-655-3p was lower in A549 and NCI-H23 cells than in BEAS2B cells (Figure 1b). These results indicate that EBLN3P and miR-655-3p may play critical roles in regulating LUAD progression.

### miR-655-3p is a direct target of EBLN3P

**Figure 2.**
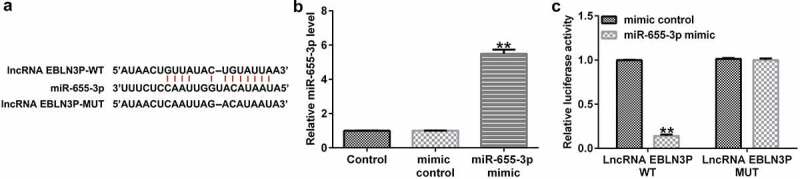
miR-655-3p is a direct target of EBLN3P.

To investigate the potential binding sites of EBLN3P and miR-655-3p, bioinformatics prediction tools (StarBase V 2.0, http://starbase.sysu.edu.cn) were used. We discovered that miR-655-3p contained potential EBLN3P binding sites (Figure 2a). Next, we conducted a dual-luciferase assay to confirm that miR-655-3p is a downstream target of EBLN3P. We confirmed that the miR-655-3p mimic notably enhanced miR-655-3p expression in HEK 293 T cells (Figure 2b). Compared with the mimic control group, the luciferase activity of the reporter plasmid containing EBLN3P-WT decreased (Figure 2c). From these results, we determined that miR-655-3p is a target of EBLN3P.

### EBLN3P negatively regulates miR-655-3p in A549 cells

**Figure 3.**
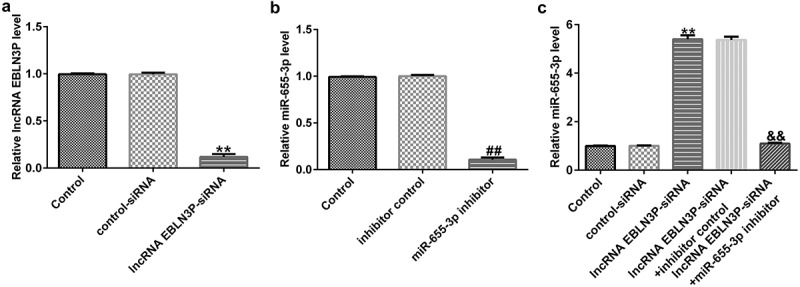
EBLN3P negatively regulated miR-655-3p expression in A549 cells.(a) The expression of EBLN3P in A549 cells upon transfection with control-siRNA or lncRNA EBLN3P-siRNA as detected via qRT-PCR. (b) The expression of miR-655-3p in inhibitor control- or miR-655-3p inhibitor transfected-A549 cells as detected via qRT-PCR. (c) The level of miR-655-3p in A549 cells was measured using qRT-PCR after transfection with control-siRNA, lncRNA EBLN3P-siRNA, lncRNA EBLN3P-siRNA + inhibitor control, or lncRNA EBLN3P-siRNA + miR-655-3p inhibitor for 48 h. **p < 0.01 vs. control-siRNA; ^##^p < 0.01 vs. Inhibitor control; ^&&^p < 0.01 vs. lncRNA EBLN3P-siRNA + inhibitor control.

To explore the effects of EBLN3P on the expression of miR-655-3p in A549 cells, A549 cells were transfected with control-siRNA, EBLN3P-siRNA, inhibitor control, miR-655-3p inhibitor, EBLN3P-siRNA + inhibitor control, or EBLN3P-siRNA + miR-655-3p inhibitor for 48 h. As shown in Figure 3a, the expression level of EBLN3P was significantly reduced in EBLN3P-siRNA-transfected cells compared to that in the control siRNA group. In addition, compared to the inhibitor NC group, the expression of miR-655-3p was lower in miR-655-3p inhibitor-transfected A549 cells (Figure 3b). As shown in Figure 3c, lncRNA EBLN3P-siRNA significantly increased miR-655-3p levels in A549 cells, while the miR-655-3p inhibitor counteracted the elevated mRNA levels of miR-655-3p.

### miR-655-3p is involved in A549 cell proliferation by mediating EBLN3P expression

**Figure 4.**
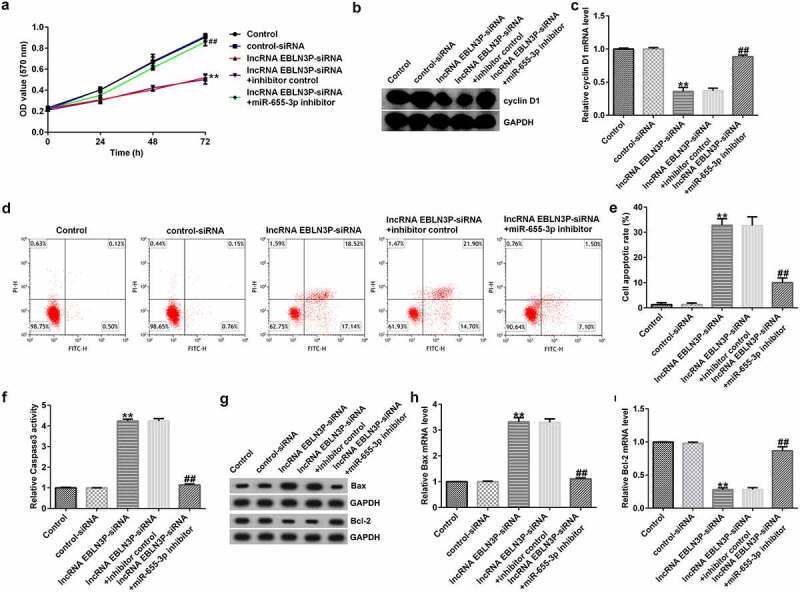
EBLN3P silencing inhibited cell proliferation and induced apoptosis in lung cancer cells by upregulating miR-655-3p.

Next, we aimed to confirm whether miR-655-3p is involved in the function of EBLN3P in lung adenocarcinoma. A549 cells were transfected with control-siRNA, lncRNA EBLN3P-siRNA, lncRNA EBLN3P-siRNA + inhibitor control, or lncRNA EBLN3P-siRNA + miR-655-3p inhibitor for 48 h. MTT analysis revealed that the viability of A549 cells transfected with lncRNA EBLN3P-siRNA decreased significantly compared to that of the control-siRNA group, while co-transfection with miR-655-3p inhibitor significantly enhanced cell proliferation (Figure 4a). Cyclin D1 is an important regulator of the cell cycle and plays a central role in cancer pathogenesis that determines uncontrolled cell proliferation [38,39]. To explore whether cyclin D1 is involved in the regulation of EBLN3P-siRNA on A549 cell proliferation, we then investigated the cyclin D1. The findings indicated that EBLN3P-siRNA repressed the mRNA and protein expression levels of cyclin D1 (Figure 4b-c). Cell apoptosis analysis revealed that EBLN3P silencing increased the apoptotic ratio of A549 cells (Figure 4d-e). We next verified the effect of lncRNA EBLN3P-siRNA on caspase-3 activity. The results showed that silencing EBLN3P significantly increased caspase-3 activity (Figure 4f). In addition, we found that EBLN3P-siRNA upregulated the protein and mRNA levels of Bax and inhibited the protein and mRNA expression of Bcl-2 (Figure 4g-i). However, treatment with a miR-655-3p inhibitor reversed the enhanced protein and mRNA levels of Bax and rescued the repressed protein and mRNA levels of cyclin D1 and Bcl-2 induced by lncRNA EBLN3P-siRNA (Figure 4b, c, g-i).

### miR-655-3p binds to Bcl-2

**Figure 5.**
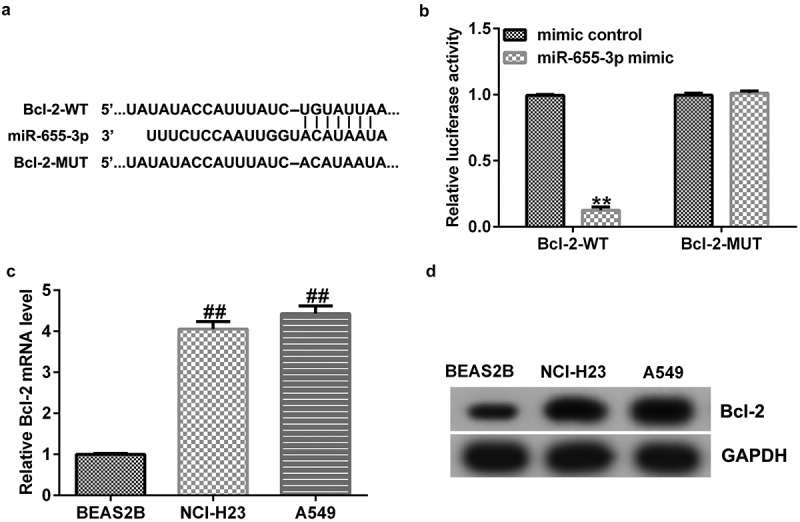
Bcl-2 is a direct target of miR-655-3p.

To explore the potential mechanisms of miR-655-3p in lung adenocarcinoma, the TargetScan database was used to predict the binding sites of miR-655-3p on Bcl-2. The results showed that Bcl-2 harbored potential miR-655-3p binding sites (Figure 5a). This prediction was verified through a dual-luciferase reporter assay. The data showed that treatment with miR-655-3p mimics decreased the luciferase fluorescence of Bcl-2-WT but had no effect on the luciferase fluorescence of Bcl-2-MUT (Figure 5b). Furthermore, according to the western blotting and qRT-PCR results, the expression level of Bcl-2 in A549 and NCI-H23 cells was higher than that in BEAS2B cells (Figure 5c-d). These results indicate that Bcl-2 is a downstream effector of miR-655-3p and its expression is negatively associated with miR-655-3p in A549 and NCI-H23 cells.

### Bcl-2 is negatively regulated by miR-655-3p in A549 cells

**Figure 6.**
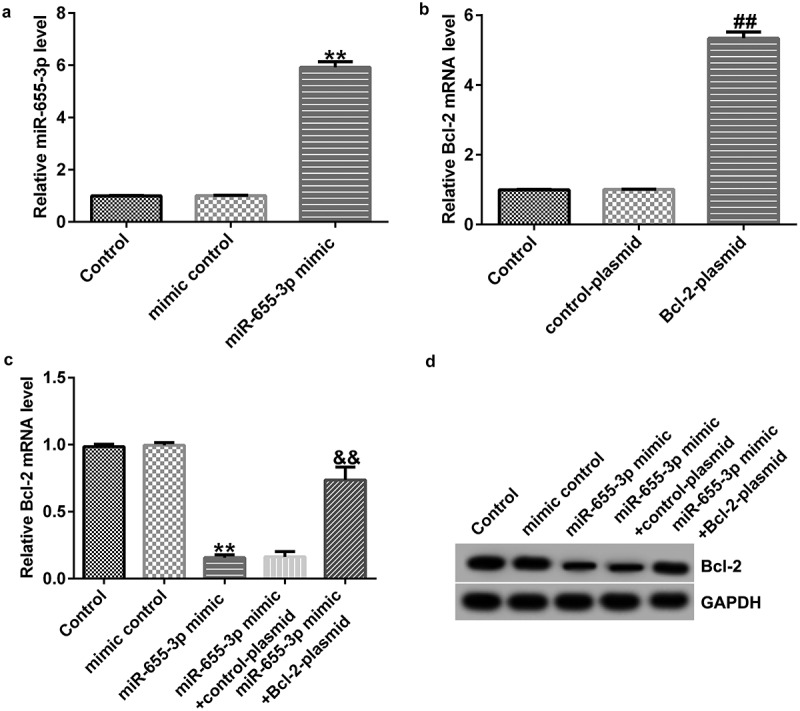
miR-655-3p negatively regulated Bcl-2 expression in A549 cells.

To further elucidate the relationship between miR-655-3p and Bcl-2, we detected Bcl-2 expression in A549 cells co-transfected with mimic control, miR-655-3p mimic, control-plasmid, Bcl-2-plasmid, miR-655-3p mimic + control-plasmid,or miR-655-3p mimic + Bcl-2-plasmid for 48 h. In contrast to the mimic control group, the miR-655-3p mimic increased miR-655-3p expression (Figure 6a). Moreover, Bcl-2 expression was higher in A549 cells after Bcl-2-plasmid transfection (Figure 6b). The protein and mRNA levels of Bcl-2 were downregulated in miR-655-3p mimic-transfected A549 cells, but they were rescued by Bcl-2-plasmid co-transfection (Figure 6c-d). These data demonstrated that miR-665-3p negatively regulates Bcl-2 expression in A549 cells.

### miR-655-3p reduces the viability and promotes the apoptosis of A549 cells via Bcl-2

**Figure 7.**
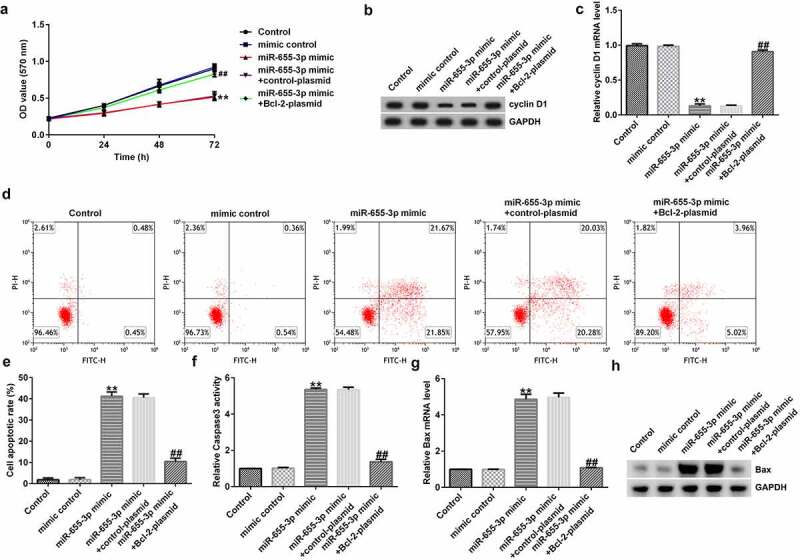
miR-655-3p inhibits the proliferation of A549 cells and induces apoptosis by downregulating Bcl-2 expression.

To explore whether miR-655-3p affected A549 cells through BCL-2, A549 cells were transfected with mimic control, miR-655-3p mimic, miR-655-3p mimic + control-plasmid, or miR-655-3p mimic + BCL-2-plasmid for 48 h. The MTT assay revealed a notable decrease in cell viability in the miR-655-3p mimic group compared to the mimic NC group, whereas cell viability was increased after transfection with a miR-655-3p inhibitor (Figure 7a). Furthermore, western blotting and qRT-PCR data showed that miR-655-3p overexpression markedly decreased cyclin D1 levels, whereas transfection with the Bcl-2-plasmid increased cyclin D1 levels in A549 cells (Figure 7b-c). Apoptosis analysis showed that the miR-655-3p mimic increased apoptosis in A549 cells, while co-transfection with the Bcl-2 plasmid significantly eliminated this effect (Figure 7d-e). In addition, we examined the effect of miR-655-3p on the activity of caspase-3 and revealed that the miR-655-3p mimic significantly increased caspase-3 activity (Figure 7f). Moreover, our data showed that miR-655-3p increased the mRNA and protein expression of Bax (Figure 7g-h) and eliminated the Bcl-2 expression (Figure 6c-d). However, co-transfection with the Bcl-2-plasmid reversed the upregulation of Bax upon miR-655-3p expression. In contrast, the Bcl-2-plasmid rescued the levels of cyclin D1 and Bcl-2 induced by the miR-655-3p mimic. Based on these results, we conclude that miR-655-3p acts as a critical suppressor in lung cancer cells proliferation through the downregulation of Bcl-2.

## Discussion

LUAD is a type of lung cancer with a persistently high morbidity rate in patients [40]. Most patients with lung adenocarcinoma are usually diagnosed at a terminal stage and have a poor prognosis. Prompt diagnosis is key to promoting remission in patients with LUAD. Although early diagnosis can be improved through evolving technology and newly developed therapies, the recurrence rate of LUAD remains unsatisfactory [4,41]. Therefore, there is a need to identify early diagnostic markers for LUAD to facilitate early detection and timely treatment.

Based on previous reports, miR-655-3p levels are reduced in non-small cell lung cancer (NSCLC) and transfection with miR-655-3p mimics hinders cell metastasis *in vitro* [18]. Another study showed that the low expression of miR-655-3p is closely correlated with positive microvascular invasion, advanced tumor stage and lymph node metastasis [42]. As expected, we determined that the expression of miR-655-3p was reduced in LUAD cells, affecting cell proliferation and apoptosis.

Recent studies have revealed that lncRNAs are new diagnostic and prognostic biomarkers for various tumors including LUAD [9,11–15,43,44], and a high expression of the lncRNA EBLN3P is associated with tumor progression [16,17,45]. Xu et al. indicated that lncRNA EBLN3P promotes colorectal cancer progression by sponging miR-323a-3p [16]. Dai et al. reported that lncRNA EBLN3P promotes the progression of osteosarcoma through modifying the miR-224-5p/Rab10 signaling axis [17]. In this study, we identified EBLN3P as a novel lung adenocarcinoma-related biomarker. Our results showed that EBLN3P was overexpressed in LUAD cell lines (A549 and NCI-H23 cells) and was negatively correlated with miR-655-3p expression. As lncRNAs exert their biological function through competing endogenous RNA (ceRNA) as miRNAs, we hypothesized that miR-655-3p might be a downstream target of EBLN3P. Our bioinformatics analysis supports our hypothesis regarding the interaction between EBLN3P and miR-655-3p, as confirmed by the results of the dual-luciferase reporter assay. Further experiments showed that EBLN3P silencing repressed the viability of LUAD cells and enhanced their apoptosis rate upon the upregulation of miR-655-3p. miRNAs can affect the levels of their target mRNAs and are involved in many biological processes [46]. To examine the mechanisms of EBLN3P and miR-655-3p associated with LUAD, we then determined the target genes of miR-655-3p. Using bioinformatics tools, we found a direct binding site between miR-655-3p and Bcl-2. Bcl-2 encodes a complete mitochondrial outer membrane that can block apoptosis in various cells [47]. An imbalance in BCL-2 expression has been reported to promote the prognosis of many types of mature NHL [48]. Moreover, Bcl-2 is related to the poor prognosis of primary central nervous system diffuse large B-cell lymphoma and breast cancer [28]. In the present study, Bcl-2 expression was upregulated in LUAD cells. These results indicate that EBLN3P promotes lung adenocarcinoma progression via the miR-655-3p/Bcl-2 axis. Although we identified a new downstream regulatory mechanism mediated by EBLN3P in lung adenocarcinoma cells, further studies are needed to clarify its underlying mechanisms. We did not investigate the relationship between lncRNA EBLN3P expression and the severity of LUAD, and the relationship between the expression of lncRNA EBLN3P in LUAD cell lines and the metastatic potential of LUAD cell lines. This was a limitation of this study, and we will study this in the next research. Moreover, in the future, it is necessary to verify the association of EBLN3P and the miR-655-3p/Bcl-2 signaling axis through EBLN3P rescue experiments and mouse models to demonstrate this new potential mechanism as a clinical therapeutic strategy.

This study is the first to prove the roles and underlying mechanisms of EBLN3P in lung adenocarcinoma. We found that EBLN3P regulates the proliferation of lung adenocarcinoma cells through the miR-655-3p/Bcl2 axis. Hence, this study provides robust evidence for identifying new biological targets for the pre-diagnosis of LUAD progression.

## Conclusion

Silencing the expression of the lncRNA EBLN3P inhibits lung adenocarcinoma cell proliferation and promotes apoptosis by regulating the miR-655-3p/Bcl-2 signaling axis. Therefore, silencing EBLN3P could be a potential therapeutic strategy for lung adenocarcinoma.

## Supplementary Material

Supplemental Material

## Data Availability

The datasets used and/or analyzed during the current study are available from the corresponding author on reasonable request.

## Research highlights

EBLN3P negatively regulates miR-655-3p in A549 cells;

miR-655-3p is involved in A549 cell proliferation by mediating EBLN3P expression;

miR-655-3p reduces the viability and promotes the apoptosis of A549 cells via Bcl-2.
