# Supplementary material for: The biological function of the long non-coding RNA endogenous born avirus-like nucleoprotein in lung adenocarcinoma is mediated through the microRNA-655-3p/B-cell lymphoma-2 axis
Source: Bioengineered. 2022 Apr 27;13(4):10679–90. doi: 10.1080/21655979.2022.2065946 (PMC9208490; doi:10.1080/21655979.2022.2065946)
Supplement: Supplemental Material [file KBIE_A_2065946_SM8359.zip › supplementary/downloadFromZipFile.pdf]

# CERTIFICATE OF ENGLISH EDITING

This document certifies that the paper listed below has been edited to ensure that the language is clear and free of errors. The edit was performed by professional editors at Editage, a division of Cactus Communications, in cooperation with Taylor & Francis Group. The intent of the author's message was not altered in any way during the editing process. The quality of the edit has been guaranteed, with the assumption that our suggested changes have been accepted and have not been further altered without the knowledge of our editors.

## Title

The biological function of the long non-coding RNA EBLN3P in lung adenocarcinoma is mediated through the miRNA-655-3p/BCL-2 axis

## Authors

Xiaopeng Wang, Jing Yin

## Order No.

VXIRZ_1

EDITINGSERVICES
Supporting Taylor & Francis authors

Signature

Vikas Narang

Vikas Narang,
Chief Operating Officer,
Editage

INFORMATION SECURITY MANAGEMENT SYSTEM DNV·GL ISO/IEC 27001

editage

Date of Issue
**February 18, 2022**
