# Supplementary material for: The biological function of the long non-coding RNA endogenous born avirus-like nucleoprotein in lung adenocarcinoma is mediated through the microRNA-655-3p/B-cell lymphoma-2 axis
Source: Bioengineered. 2022 Apr 27;13(4):10679–90. doi: 10.1080/21655979.2022.2065946 (PMC9208490; doi:10.1080/21655979.2022.2065946)
Supplement: Supplemental Material [file KBIE_A_2065946_SM8359.zip › supplementary/Supplementary material.zip!/Supplementary material/Original blots of Western blot assay/Note.docx]

Dear editor,

Thank you very much for your consideration of our manuscript. In the file are original blots of all proteins. And it should be noted that during the Western blot assay, we first cut out the corresponding membrane according to the molecular weight of the target protein and then incubate the primary antibody. Therefore our original imprint is not a full film. We guarantee that all our experiments are carried out scientifically and strictly in accordance with the experimental procedures. Hope to get your understanding and support.

Best regards
